# Protocol for whole-brain immunolabeling, clearing, and light-sheet imaging of C-FOS in the pigeon

**DOI:** 10.1016/j.xpro.2026.104462

**Published:** 2026-03-24

**Authors:** Spencer D. Balay, Gregory C. Nordmann, Simon Nimpf, E. Pascal Malkemper, Lukas Landler, David A. Keays

**Affiliations:** 1 Department of Biology, Ludwig-Maximilians-University Munich, Planegg-Martinsried, 82152 Planegg, Germany; 2 Max Planck Institute for Biological Intelligence, Planegg-Martinsried, 82152 Planegg, Germany; 3 Max Planck Institute for Neurobiology of Behavior - Caesar, 53175 Bonn, Germany; 4 BOKU University, Institute of Zoology, Department of Ecosystem Management, Climate and Biodiversity, 1180 Vienna, Austria; 5 University of Cambridge, Department of Physiology, Development & Neuroscience, CB2 3EG Cambridge, UK; 6 Research Institute of Molecular Pathology (IMP), Vienna Biocenter (VBC), 1030 Vienna, Austria

**Keywords:** Model Organisms, Neuroscience, Systems biology

## Abstract

Advancements in tissue clearing and light sheet microscopy have provided new ways to study neural circuits dedicated to sensory processing in a diverse range of animal species. Here, we present a modified iDISCO+ protocol for whole-brain immunolabeling with the neural activity marker C-FOS in the pigeon brain. We describe steps for whole-brain pre-processing, bleaching, and immunostaining. We then detail procedures for obtaining ultramicroscopic light-sheet images in cleared pigeon brains.

For complete details on the use and execution of this protocol, please refer to Nordmann et al.^1^

## Highlights

• Optimized whole-brain clearing protocol for avian brain tissue
• Techniques for utilizing the neuronal activity marker C-FOS after sensory stimulation
• Steps for light-sheet imaging and visualization of C-FOS in the whole pigeon brain

## Abstract (editor highlights)

Publisher’s note: Undertaking any experimental protocol requires adherence to local institutional guidelines for laboratory safety and ethics.

## Graphical abstract

**Figure:**
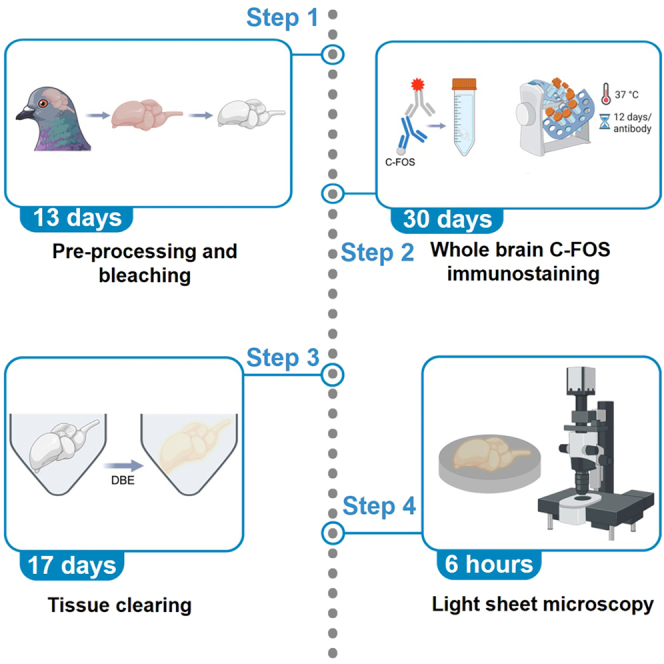

## Before you begin

This protocol describes an optimized C-FOS immunolabeling and clearing procedure for the pigeon (*Columba livia*) brain. The use of solvent-based clearing techniques such as iDISCO,^2^ CUBIC^3^ and CLARITY^4^ has increased considerably over the last decade. These methods reduce heterogenous light scattering in tissue samples through dehydration, delipidation, and refractive index homogenization, all while permitting immunolabeling of activity markers.^5^^,^^6^ Light sheet microscopy is an ideal approach to image cleared brains. This optical sectioning technique allows fast and high-resolution 3D volumetric imaging of the fully intact brain sample.^7^^,^^8^ Associated software pipelines such as ClearMap count labeled cells, map each brain to a standardized reference atlas, and identify anatomical regions with increased neural activity.^9^ These techniques have been used in systems neuroscience to uncover the neural circuits involved in food intake control,^10^ maternal preparatory nesting behavior,^11^ opioid reward,^12^ and avian magnetoreception.^1^

### Innovation

Although tissue clearing is a widely used technique, protocols specifically optimized for avian brains are limited.^13^^,^^14^ To rectify this, we outline a refined iDISCO^+^ protocol that enables high-resolution imaging of the neural activity marker C-FOS in cleared pigeon brains. We provide optimal solution incubation times for dehydration, bleaching, immunolabeling, delipidation, and refractive index matching. Additionally, we describe a light sheet imaging strategy that can be uniformly adapted to avian brains. Using this method, we have mapped established auditory centers in the avian brain and have uncovered novel neuronal circuits dedicated to the processing of magnetic information.^1^ This versatile protocol can be used to probe multiple sensory or behaviorally related questions in birds, such as the neuronal circuits dedicated to vision, olfaction, mechanosensation, or cognition.

### Institutional permissions

Animal housing and experimental procedures were performed in accordance with existing ethical frameworks by the City of Vienna (GZ: 214635/2015/20 and GZ: 256884/2020) and the Regierung of Oberbayern (ROB-55.2-2532. Vet_02-21-86).

## Key resources table

**Table:** 

REAGENT or RESOURCE	SOURCE	IDENTIFIER
**Antibodies**		

Donkey anti-Mouse Alexa Fluor 647 (1:300)	Thermo Fisher Scientific	A-31571
Mouse monoclonal anti-C-FOS (E−8) antibody (1:300)	Santa Cruz Biotechnology, Inc.	sc-166940

**Chemicals, peptides, and recombinant proteins**		

10x PBS (pH 7.4)	N/A; in-house.	See recipe below.
Dibenzylether	Sigma-Aldrich	33630-1L
Dichloromethane ≥99.5%, for synthesis	Carl Roth	8424.1
DMSO	Sigma-Aldrich	276855
Donkey Serum – Sterile, not heat-inactivated.	Biowest	S2170 - 500
Glycine	AppliChem	A1067.5000
Heparin sodium salt	Carl Roth	7692.2
Hydrogen Peroxide (30%)	VWR Chemicals	8.22287.1000
Methanol ≥99.5%, GPR RECTAPUR	VWR Chemicals	20846.326
Paraformaldehyde	Carl Roth	0335.3
Sodium azide, NaN_3_	Sigma-Aldrich	S2002
Triton X-100	Sigma-Aldrich	X100
Tween 20	Sigma-Aldrich	P9416

**Experimental models: Organisms/strains**		

*Columba livia:* Wildtype strain	N/A	N/A

**Software and algorithms**		

ClearMap 1.0	Renier et al., 2016^2^	https://github.com/ChristophKirst/ClearMap
ImSpector 5.1.351	Miltenyi Biotec	N/A

**Other**		

50 mL centrifuge tube racks	N/A	N/A
Bottle top filters 0.2 μm	Thermo Fisher Scientific	595–4520
Diamond engraving pen	Carl Roth	1530.1
50 mL Falcon centrifuge tubes	Corning	352070
Glass vials 10 ml (45 mm height)	Carl Roth	X655.1
Incubator	Thermo Fisher Scientific	51028132
Lightsafe 50 mL centrifuge tubes	Sigma-Aldrich	Z688339-50EA
Parafilm	VWR International	291–1213
Pigeon brain sample holder	N/A; in house.	https://github.com/KeaysLab/ClearingProtocol
Shaker	Heidolph	543-42210-00
Staining troughs, with lid	VWR International	BRND472200
Ultramicroscope II	Miltenyi BioTec	N/A

## Materials and equipment

**CRITICAL:** Reagents should be prepared fresh on the day of use. This is especially important for the permeabilization, blocking, and staining solutions that contain serum. Here, we provide the experimental day when we recommend preparing reagents. Specific amounts of each solution will depend on the sample size.

**Table:** 10× PBS (Day 0 – day of perfusion)

Reagent	Final concentration	Amount
NaCl	1.37 M	80 g
KCl	27 mM	2 g
Na_2_HPO_4_ · 2H_2_O	100 mM	17.8 g
KH_2_PO_4_	18 mM	2.4 g
MilliQ H_2_O	N/A	1000 mL
**Total**	N/A	1000 mL

Adjust pH to 7.4. Store at room temperature (20°C–25°C). Max storage time: Use within 6 months.

**Table:** 1× PBS- Heparin (Day 0 – day of perfusion)

Reagent	Final concentration	Amount
20X Stock Heparin (240 KU/L) in 1× PBS	1X	50 mL
10× PBS	1X	100 mL
MilliQ H_2_O	N/A	850 mL
**Total**	N/A	1000 mL

Filter sterilize with a 0.2 μm bottle top filter. Store at 4°C. Warm to 37°C immediately before use. Max storage time: Prepare fresh, use within 2 weeks.

**Table:** 4% Paraformaldehyde (PFA) in 0.2M phosphate buffer (Day 0 – day of perfusion)

Reagent	Final concentration	Amount
0.2 M NaH_2_PO_4_ (24 g in 1 L MilliQ water)	0.2 M	100 mL
0.2 M Na_2_HPO_4_ (28.48 g in 1 L MilliQ water)	0.2 M	400 mL
MilliQ H_2_O	N/A	500 mL
Paraformaldehyde (PFA) powder	4%	40 g
**Total**	N/A	1000 mL

To aid PFA dissolving, first heat phosphate buffers and 300 mL of MilliQ water to 70°C and then add PFA powder. Dissolve on a magnetic stirrer at 70°C. Top up to 1 liter with MilliQ water, filter sterilize with a 0.2 μm bottle top filter and store at 4°C. Warm to room temperature before perfusion. Max storage time: Prepare fresh, use within 2 weeks.

**Table:** 5% H_2_O_2_ in methanol (Day 7 – day of bleaching)

Reagent	Final concentration	Amount
30% H_2_O_2_	5%	167 mL
Methanol	N/A	833 mL
**Total**	N/A	1000 mL

Make sure solution is pre-chilled to 4°C before use. Max storage time: Prepare fresh, use within 2 weeks.

**Table:** PT×.2 (Day 14 – day of permeabilization)

Reagent	Final concentration	Amount
Triton X-100	0.2%	2 mL
10× PBS	1×	100 mL
MilliQ H_2_O	N/A	898 mL
**Total**	N/A	1000 mL

Store at room temperature (20°C–25°C). Max storage time: Prepare fresh, use immediately.

**Table:** Permeabilization solution (Day 14 – day of permeabilization)

Reagent	Final concentration	Amount
PT×.2	N/A	800 mL
DMSO (100%)	20%	200 mL
Glycine	N/A	23g
Sodium azide NaN_3_ (10%)	0.02%	2 mL
**Total**	N/A	1000 mL

Filter sterilize with a 0.2 μm bottle top filter. Prepare fresh. Warm to 37°C immediately before use. Max storage time: Prepare fresh, use immediately.

**Table:** Blocking solution (Day 16 – day of blocking)

Reagent	Final concentration	Amount
PT×.2	N/A	840 mL
DMSO (100%)	10%	100 mL
Donkey Serum (100%)	6%	60 mL
Sodium azide NaN_3_ (10%)	0.02%	2 mL
**Total**	N/A	1000 mL

Filter sterilize with a 0.2 μm bottle top filter. Prepare fresh. Warm to 37°C immediately before use. Max storage time: Prepare fresh, use immediately.

**Table:** PTwH (Day 18, 30, 31 and 43 – days of antibody staining and rinsing)

Reagent	Final concentration	Amount
10× PBS	1×	100 mL
MilliQ H_2_O	N/A	897 mL
Tween-20	0.2%	2 mL
Heparin (10 mg/mL)	0.1 M	1 mL
Sodium azide NaN_3_ (10%)	0.02%	2 mL
**Total**	N/A	1000 mL

Filter sterilize with a 0.2 μm bottle top filter. Store at room temperature (20°C–25°C). Max storage time: Prepare fresh, use immediately.

**Table:** Primary antibody solution (Day 18 – day of primary antibody incubation)

Reagent	Final concentration	Amount
PTwH	N/A	275 mL
DMSO (100%)	5%	15 mL
Donkey Serum (100%)	3%	9 mL
Mouse-anti-C-FOS (E−8)	1:300	1 mL
Sodium azide NaN_3_ (10%)	0.02%	2 mL
**Total**	N/A	300 mL

Filter sterilize with a 0.2 μm bottle top filter. Change volume depending on sample size. Max storage time: Prepare fresh, use immediately.

**Table:** Secondary antibody solution (Day 31 – day of secondary antibody incubation)

Reagent	Final concentration	Amount
PTwH	N/A	290 mL
Donkey Serum (100%)	3%	9 mL
Donkey-anti-Mouse Alexa Fluor 647	1:300	1 mL
Sodium azide NaN_3_ (10%)	0.02%	2 mL
**Total**	N/A	300 mL

Filter sterilize with a 0.2 μm bottle top filter. Change volume depending on sample size. Max storage time: Prepare fresh, use immediately.

## Step-by-step method details

### Sensory stimulation, perfusion, and brain isolation—Day 0

**Timing: 2–3 h**

This step describes how to isolate adult pigeon brains after a neuronal activation paradigm.

1. Expose animals to sensory stimulation.

***Note:*** Immediate early gene products such as C-FOS protein peak 1 to 2 h after exposure to a stimulus.^15^ Therefore, we recommend stimulating animals continuously for at least 1 h.

2. After stimulation, immediately sacrifice the animal and perfuse transcardially with PBS/PFA.
  a. Open the body cavity and release the heart from the pericardium.
  b. Make an incision in the right auricle.
  c. Perfuse 200 mL of 1× PBS-Heparin (12U/L) warmed to 37°C into the left ventricle of the heart using a 23 G needle at a flow rate of 25 mL/min.
  d. Change the perfusion solution to room temperature 4% PFA and perfuse with 200 mL of fixative.

***Note:*** Unless required by the approved animal protocol, avoid pre-sacrifice sedation because of possible effects on C-FOS expression.

3. Carefully dissect the brain from the skull cavity in 1x PBS.
  a. Make sure to fully remove the dura mater to facilitate proper antibody penetration.
  b. Fully resect the optic chiasm at its base to reduce tissue size.
  c. Open the cisterna magna over the brain stem to avoid formation of air bubbles during final clearing steps.
  d. Leave approximately 1 cm of the spinal cord attached to facilitate sample handling and transfer.
4. Transfer each brain to a 50 mL Falcon tube and post-fix in 25 mL of 4% PFA at 4°C for 20 h without rotation.
5. Remove PFA the next day and add 50 mL of 1x PBS cooled to 4°C. Store brains at 4°C until the experiment is complete.

**CRITICAL:** Fixation time should remain the same between treatment groups when comparing immediate early gene protein levels. Additionally, over-fixation can lead to excessive cross-linking and insufficient immunolabeling.

**Pause Point:** For long-term storage (>1 month, max. 2 months) of pigeon brains, add 0.02% Sodium azide to PBS to prevent microbial growth and store at 4°C. We recommend processing the brains as soon as possible.

### Pre-staining dehydration—Day 1

**Timing: 7 days**

This step dehydrates the brains in preparation for immunostaining.

**CRITICAL:** Dilute methanol in 1× PBS. This prevents tissue swelling and rupture during dehydration and rehydration compared to diluting methanol in H_2_O. Handle methanol in a chemical hood and dispose according to local safety regulations.

***Note:*** For each methanol step, perform two rinses per day (approximately 12 h apart; 10% increments per wash); e.g.one in the morning, and one at the end of the day before leaving for overnight incubation. All steps are conducted at room temperature, shaking at 20 rpm unless noted otherwise. Use a resistant labeling method (such as diamond pens) to prevent washout of sample IDs on tubes.

6. Dehydrate brains in 10% increments of methanol/1× PBS.
  a. Transfer 50 mL of fresh 1× PBS cooled to 4°C and shake tubes gently (20 rpm) at room temperature until they are warmed to 20°C–25°C.
  b. Remove 1× PBS and add 50 mL of 10% methanol/1× PBS to each tube. Shake overnight.
  c. The next morning, remove 10% methanol/1× PBS and add 20% methanol/1× PBS.
  d. Repeat in 10% increments until 80% methanol/1× PBS is reached.
    ***Note:*** The 90% methanol/1× PBS step is skipped because we have observed precipitate formation while preparing this solution. To avoid tissue damage, handle tubes containing dehydrated samples with care during solution exchange.
  e. Wash two times in 100% methanol over a day at room temperature.
  f. Remove 100% methanol and add 66% Dichloromethane (DCM)/methanol. Fill tubes to the top (>50 mL) to prevent oxidation and shake overnight.
  g. The next day, remove 66% DCM/methanol and rinse with 100% methanol thrice, 1 h each at room temperature.
  h. During the third and final methanol wash, put the brains onto a shaker at 4°C until the samples are cooled down (∼4 h).
    **CRITICAL:** Handle Dichloromethane (DCM) in a chemical fume hood and dispose according to local safety regulations. DCM can easily erode common plastic labware and requires careful handling. Fill tubes completely to prevent oxidation. Store secured in a chemical cabinet.

### Bleaching—Day 7

**Timing: 2 days**

This step reduces background by quenching innate autofluorescence of brain tissue and autofluorescence due to paraformaldehyde fixation.

7. Bleaching.
  a. Remove methanol and add cooled 5% H_2_O_2_/methanol to each tube.
  b. Shake gently at 4°C for 48 h. The brains should appear fully white after bleaching properly.
  c. After two days, remove 5% H_2_O_2_/methanol and add 100% methanol cooled to 4°C.
  d. After transferring the cooled methanol, perform this wash at room temperature (∼2 h) to gradually warm the brains up.
  e. Add fresh room temperature 100% methanol and wash overnight on a shaker.

**CRITICAL:** Handle H_2_O_2_ in a chemical fume hood and dispose according to local safety regulations.

### Pre-staining rehydration—Day 10

**Timing: 4 days**

This step rehydrates the brains in preparation for immunostaining.

8. Rehydrate brains in 10% steps of methanol/1× PBS.
  a. Remove 100% methanol and rehydrate with 80% methanol/1× PBS.
  b. Rehydrate in 10% steps of methanol/1× PBS (2 steps/day) until 20% methanol/1× PBS is reached.
  c. Remove 20% methanol/1× PBS and rinse in 1× PBS overnight at room temperature on a shaker.

### Whole-brain immunostaining—Day 14

**Timing: 30 days**

***Note:*** Addition of 0.02% sodium azide to solutions that are used at 37°C for multiple days is highly recommended! This includes Permeabilization, Blocking and Primary/Secondary antibody solutions.

9. Permeabilization (2 days).
  a. Remove 1× PBS and rinse twice (3 h each) in PT×.2 solution.
  b. Add freshly prepared, 0.2 μm sterile-filtered Permeabilization solution and incubate the brains at 37°C on a shaker for 48 h.
10. Blocking (2 days).
  a. After two days, remove Permeabilization solution and add pre-warmed, sterile-filtered Blocking solution.
  b. Incubate brains at 37°C on a shaker for 48 h.
    ***Note:*** To preserve reagents, antibody incubations are performed in 10 mL glass vials.

11. Primary antibody incubation (12 days).
  a. Prepare sterile-filtered PTwH solution with 5% DMSO and 3% donkey serum, and prewarm to 37°C.
  b. Engrave sample ID with diamond pen on 10 mL glass vials.
  c. Rinse empty vials once with PTwH/5% DMSO/3% donkey serum just prior to transferring brains out of blocking solution.
  d. Discard blocking solution and transfer individual brains to the appropriate 10 mL vial.
  e. Fill with 10 mL of primary antibody solution (mouse anti-C-FOS, 1:300 in PTwH/5% DMSO/3% donkey serum).
  f. Save 10 mL of solution without antibody for the negative control brain.
  g. Wrap the lid of each glass vial with parafilm to prevent contamination during the long-term incubation.
  h. Incubate the brains for 12 days at 37°C on a rotating shaker.
  i. Individual glass vials are contained within a sterile box that is also wrapped with parafilm.
12. PTwH Washes (1 day).
  a. Remove primary antibody solution and store at 4°C.
  b. Transfer the brains to fresh 50 mL Falcon tubes and fill with prewarmed PTwH.
  c. Wash for 1 h at 37°C on a shaker.
  d. Add new PTwH and wash overnight at 37°C.
  e. The next day, wash with fresh PTwH 3 times (2 h each).
13. Secondary antibody incubation (12 days).
  a. Prepare sterile-filtered PTwH/3% donkey serum, and prewarm to 37°C.
  b. Engrave sample ID with diamond pen on 10 mL glass vials.
  c. Rinse empty vials once with PTwH/3% donkey serum just prior to transferring brains out of PTwH wash solution.
  d. In a darkened room under minimal light, fill vials with 10 mL of secondary antibody solution (donkey anti-mouse Alexa Fluor 647 1:300 in PTwH/3% donkey serum).
  e. Discard wash solution.
  f. Transfer individual brains to the appropriate 10 mL vial.
  g. Wrap the lid of each glass vial with parafilm to prevent contamination during the long-term incubation.
  h. Incubate the brains for 12 days at 37°C on a rotating shaker in the dark.
  i. Contain individual glass vials within a sterile plastic pipette tip box that is also wrapped with parafilm.
14. PTwH Washes (1 day).
  a. Remove secondary antibody solution and transfer the brains to fresh light-safe 50 mL Falcon tubes and fill with prewarmed PTwH.
  b. Wash for 1 h at 37°C on a shaker.
  c. Add new PTwH and wash overnight at 37°C.
    ***Note:*** Primary antibody solution can be reused (we have seen comparable signal using solution up to 6 months old). Re-sterile filter (0.2 μm) before use.

### Tissue clearing—Day 44

**Timing: 17 days**

15. Final dehydration (9 days).
  a. Wash with fresh PTwH 3 times (2 h each).
  b. Remove PTwH and add 10% methanol/1× PBS.
  c. Shake overnight at room temperature.
  d. Dehydrate brains in 10% steps of methanol/1× PBS (2 steps/day) until 80% methanol/1× PBS is reached.
  e. Dehydrate brains in 100% methanol and wash overnight.
  f. The next day, change to fresh 100% methanol.
  g. In the evening, change solution to 66% DCM/34% methanol and wash overnight.
  h. Change solution to fresh 66% DCM/34% methanol and wash for∼12 h.
  i. Discard 66% DCM and change to fresh 100% DCM.
  j. Shake overnight at room temperature.
  k. The next day, change to fresh 100% DCM and shake at room temperature for 48 h.
    ***Note:*** The 90% methanol/1× PBS step is skipped because we have observed precipitate formation while preparing this solution. To avoid tissue damage, handle tubes containing dehydrated samples with care during solution exchange.

**CRITICAL:** Final clearing steps are performed in glass vials, as DBE can degrade plastics. Handle DBE and DCM in a chemical hood and dispose according to local safety regulations. Store secured in a chemical cabinet away from heat sources.

16. Clearing (8 days).
  a. Engrave sample ID with diamond pen on 10 mL glass vials and lid.
  b. Add 5 mL of Dibenzyl ether (DBE) to the vial.
  c. Remove 100% DCM and transfer brain into appropriate glass vial filled with 5 ml DBE.
  d. Rinse briefly and discard solution to remove residual DCM.
  e. Re-fill glass vial with 10 mL of fresh DBE and store out of light.
  f. After 3 days, change to fresh DBE.
  g. After 8 total days in DBE, samples should be sufficiently cleared for imaging.
    ***Note:*** Store individual brains within a glass staining jar with the experiment name engraved on the lid and the side of the jar in the dark prior to imaging.

### Light sheet microscopy—Day 60

**Timing: 6 h/brain**

The microscope used in this protocol is the Ultramicroscope II from Miltenyi Biotec with a MV PLAPO 2XC objective (Olympus, #PLAPON2X) with DBE dipping cap and an sCMOS camera (ANDOR Neo 5.5). Images were acquired using a 2 × 0.8 magnification, resulting in a 4.064 × 4.064 μm resolution over a field of view of 8.7 × 10.4 mm.

17. Microscope calibration and imaging chamber preparation.
  a. Clean the imaging chamber by rinsing with water and 95% ethanol.
  b. Air dry the chamber before the imaging session begins (usually overnight).
  c. Fill the imaging chamber with fresh DBE just before the fill line.
  d. Adjust the position of the light sheet beam paths using the alignment tool according to the manufacturer’s recommendations (available at https://github.com/KeaysLab/ClearingProtocol).
18. Sample trimming and mounting.
  a. Prepare the sample holder with the correct brain insert (Dorsal or Ventral mold).
  b. Fill the insert with DBE to limit the time the sample is out of the solution when mounting.
  c. Take the brain out of the sample vial and cut off the spinal cord behind the medulla with small scissors. Leave about 2 mm under the posterior end of the cerebellum.
  d. Transfer the brain to the appropriate insert and move both sliders to fix the brain in place without applying too much pressure.
  e. Tighten screws and remove all excess DBE from the sample holder with a paper towel.
    **CRITICAL:** To prevent DBE contamination from gloves being transferred to parts of the microscope, remove contaminated gloves after DBE-handling and mount the cleaned sample holder into the imaging chamber with a fresh pair of gloves.

19. Set up imaging parameters.
  a. For imaging the right hemisphere in the Dorsal orientation, put the sample holder into the imaging chamber so anterior is facing upward (away from the researcher).
  b. Adjust DBE levels so they are at the fill line.
  c. Tighten the sample holder in place with screws on the stage.
  d. Move the sample to the bottom of imaging chamber using the Z-wheel of the control dial.
  e. Activate the right set of light sheets in Imspector, as our imaging strategy images the complete right hemisphere of the brain.
  f. Switch on the 488 nm laser at 2% intensity to illuminate the autofluorescence channel and move the sample in Z until the start of the tissue is illuminated.
  g. Manually rotate the objective lens and lower into DBE using the coarse focus.
  h. Find the focal plane by adjusting the coarse focus and illumination.
  i. Once the sample is in focus, lower its position using the Z-wheel until the light sheet does not illuminate any tissue.
  j. Set this current Z-position as 0.00 in Imspector and switch off the laser.
    ***Note:*** To image all brains in a similar fashion, we have developed a landmark-based imaging strategy. The right hemisphere of each brain is imaged in Dorsal and Ventral halves. To image the left hemisphere, repeat steps 19–26 with the left hemisphere in the field of view. Use the same landmarks as described below. In step 19d, align and activate the left set of light sheets. We have found the most consistent imaging quality when using light sheets from one side.

20. Define imaging landmarks (Dorsal half).
  a. Draw a box with a width of 300 pixels and move it to the left border of the imaging window.
  b. Move to the Z-position of −3500 μm and switch on the 488 nm laser at 2% intensity.
  c. Move in Z until the dorsal start of the optic tectum is reached.
  d. Use the crosshair to align the midline of the brain (longitudinal fissure) vertically and move the sample in X until the fissure is positioned 300 pixels from the left imaging border (using the 300-pixel wide box).
  e. Move the sample in Y until the posterior end of the forebrain is aligned to the lower imaging border.
  f. Adjust the sample holder ring manually in case the sample is tilted.
    ***Note:*** To image the entire Anterior-Posterior axis, we image each hemisphere in two tiles that overlap by 20%.

21. Set the tiles and image capture settings.
  a. Under the Mosaic tab, click ‘Set Parameters’ and ensure that 20% overlap is selected.
  b. Clear any existing mosaic that was previously defined.
  c. Double click on the current position in the tile window and drag down to generate a second tile.
  d. Double click on the upper tile to check the final Y position of the sample. Switch off the laser.
  e. Move down in Z to position −4800 μm.
  f. Switch on the 488 nm laser and make sure that no lateral or anterior tissue is outside the field of view.
  g. Slowly move to position −5500 μm to check whether the objective touches the tissue (dark shadow as well as movement of the sample would be visible).
  h. Switch off the laser.
  i. Move up in Z to position −1000 μm.
  j. Position the medial hippocampus under the dynamic horizontal focus position (white arrowhead).
  k. Switch on the 640 nm laser, set to 2% intensity, and use the fine focus of the objective to get the sharpest image of C-FOS+ cells.
  l. Switch off the laser.
    ***Note:*** If −5500 μm cannot be reached due to the objective hitting the tissue, either remount the sample or change the start position of the imaging from “0” to the corresponding amount missing to reach 5500 μm total volume. For example, if the deepest imaging plane is at −5300 μm, set the imaging start position at +200 μm. This is important for downstream analyses that require the same number of images across brains.

22. Image the 640 channel with the following parameters.
  a. Measurement mode: Mosaic Acquisition.
  b. Devices: Z, Y, X, Automatic Save (AS): ON.
  c. Laser power: 100%
  d. Sheet width: 100%
  e. Sheet NA: 0.109 (thickness 7 μm).
  f. Zoom: 0.8×
  g. Light sheet: Right laser.
  h. Liquid: DBE.
  i. Dynamic Horizontal Focus (DHF): ON (16 steps).
  j. Start position: 0.000 μm, End position: −5500 μm, Range: −5500 μm, Stepsize: 5.000 μm, # Images: 1101.
  k. Exposure time: ∼200 ms.
  l. Click ‘Autosave settings’ and rename to the appropriate sample ID.
  m. Take the background image and click ‘Use background’.
  n. Start the measurement.
23. Image the 488 channel with the following parameters.
  a. Turn down laser power (22c) to 50% and set the Dynamic Horizontal Focus (22i) to OFF.
  b. Leave the remaining settings the same.
  c. Click ‘Autosave settings’ and rename to the appropriate sample ID.
  d. Take the background image and click ‘Use background’.
  e. Start the measurement.
24. Mount sample in Ventral brain insert.
  a. Remove the dorsal insert from the sample holder and flip the brain to image the ventral half.
  b. Press the ventral brain insert into sample holder ring.
  c. Move anterior sliders until they touch the sample below the olfactory bulbs. The posterior slider should hold the brain stem where the cut was made at the beginning of the imaging session.
  d. Remove any excess DBE from the sample holder ring and transfer it to the imaging chamber.
  e. To image the right hemisphere of the brain in the ventral orientation, ensure the anterior end of the sample is facing downward (pointing towards the researcher).
  f. Repeat steps 19c-j. Make sure to set a new Z position to 0.00 at the start of the tissue.
25. Define imaging landmarks (Ventral half).
  a. Move to the Z-position of −3500 μm and switch on the 488 nm laser at 2% intensity.
  b. Move in Z until the beginning of the anterior commissure.
  c. Use the crosshair to align the midline of the brain (longitudinal fissure) vertically and move the sample in X until the fissure is positioned 300 pixels from the left imaging border (using the 300-pixel wide box).
  d. Move the sample in Y until the posterior end of the septal nuclei are aligned to the horizontal line of the cross hair.
  e. Adjust the sample holder ring manually in case the sample is tilted.
26. Set the tiles and image capture settings.
  a. Clear any existing mosaic that was previously defined.
  b. Double click on the current position in the tile window and drag up to generate a second tile.
  c. Double click on the upper tile to check the final Y position of the sample. Switch off the laser.
  d. Ensure the sample can be imaged until a Z position of −5500 μm without the objective hitting the tissue.
  e. Move up to the Z position of −2500 μm.
  f. Position the olfactory bulbs under the dynamic horizontal focus position (white arrowhead).
  g. Switch on the 640 nm laser, set to 2% intensity, and use the fine focus of the objective to get the sharpest image of C-FOS+ cells.
  h. Switch off the laser.
  i. Image the sample with the same parameters as outlined in Steps 22 and 23.

***Note:*** If imaging a larger brain, or using a different objective lens and magnification, the size of the reference box (currently 300 pixels) may need to be modified. Ensure the lateral-most tissue is included in the imaging tile (not cut off).

## Expected outcomes

**Figure 1:**
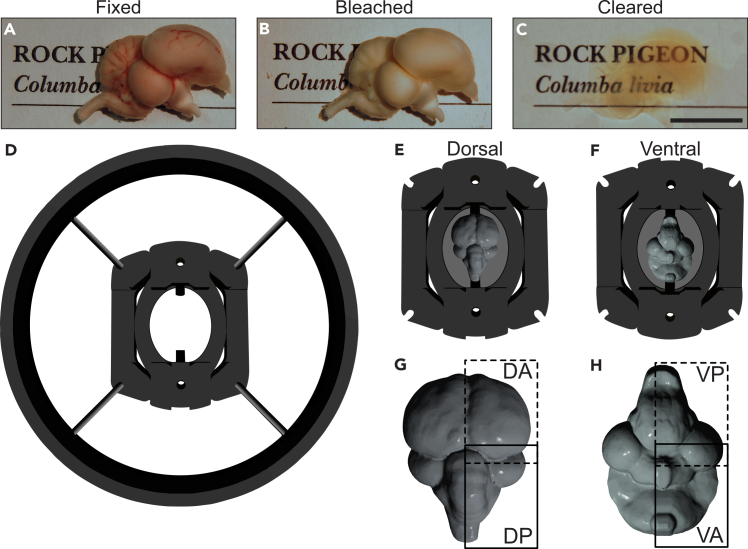
Pigeon whole brain clearing and imaging (A) Image of a perfused brain, (B) following bleaching, and (C) after delipidation. (D) Sample holder employed to image brains that fits into the DBE-filled chamber of the light sheet microscope. Brains are mounted in a (E) Dorsal or (F) Ventral 3D-printed pigeon brain mold. The complete right hemisphere is imaged along the dorsoventral axis, with two tiles along the anteroposterior axis with 20% overlap. This results in four separate imaging tiles. (G) DA: dorsal-anterior and DP: dorsal-posterior; (H) VP: ventral-posterior and VA: ventral-anterior. Scale bar shows 10 mm.

The execution of this protocol should result in 1) fully cleared pigeon brain tissue; 2) specific staining of the immediate early gene C-FOS throughout the entire brain; and 3) high-quality light sheet image stacks of individual pigeon brains. Using this optimized clearing pipeline, perfused, post-fixed, and stained pigeon brains are rendered transparent with minimal browning of the tissue (Figures 1A–1C). The samples are mounted for imaging using a custom pigeon brain sample holder and pigeon brain mold (Figures 1D–1F). The complete right-hemisphere of each brain is imaged along the dorsal-ventral axis, in two tiles that overlap by 20%. This results in four image files being created for each plane in a single brain: Dorsal-Anterior (DA), Dorsal-Posterior (DP), Ventral-Anterior (VA), and Ventral-Posterior (VP) (Figures 1G and 1H).

**Figure 2:**
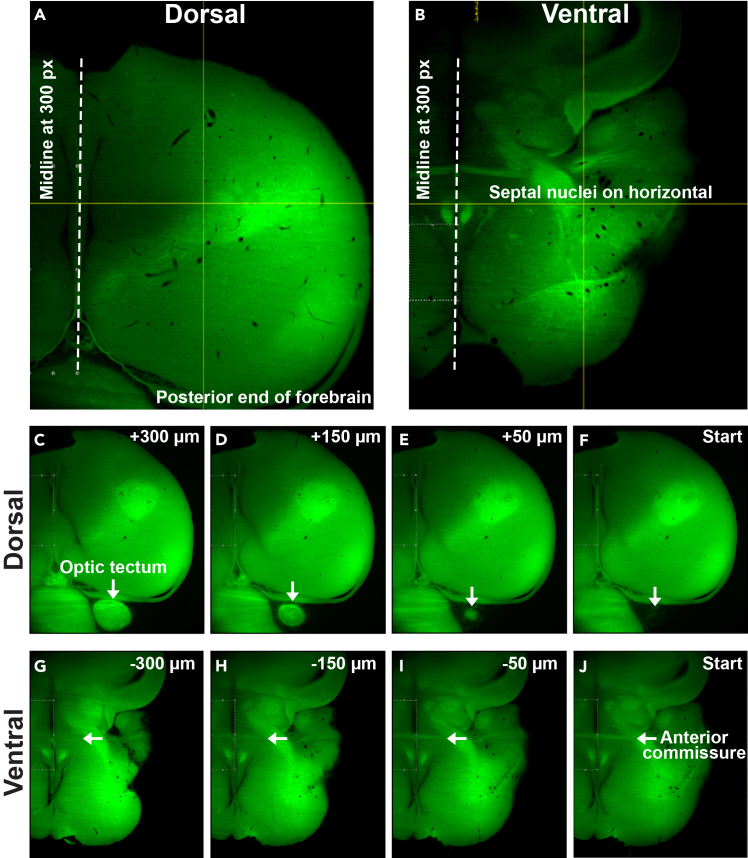
Imaging strategy and anatomical landmarks (A) Dorsal and (B) Ventral anatomical landmarks for imaging the right hemisphere of cleared pigeon brains. (C–F) Sequence of images depicting the dorsal start of the optic tectum Z-plane (panel F). Panel C shows 300 μm passed the landmark frame in the Z-plane. (G–J) Sequence of images depicting the start of the anterior commissure Z-plane (panel J). Panel G shows 300 μm before the landmark frame in the Z-plane.

**Figure 3:**
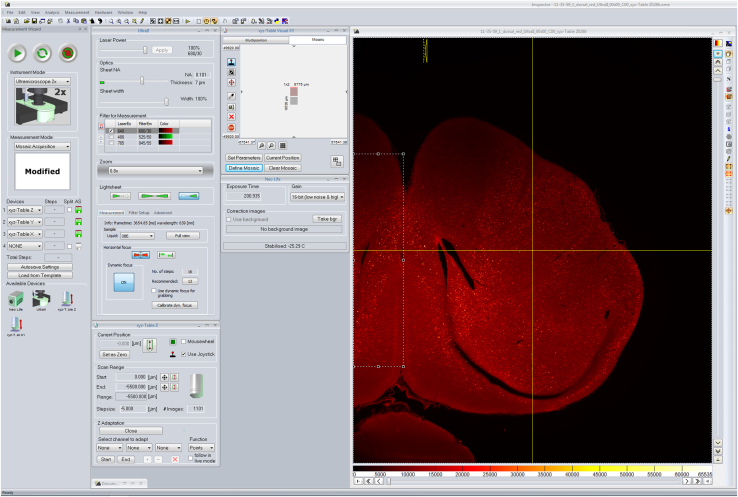
Imspector settings for acquiring light sheet images Ensure the following settings are set: Measurement mode: Mosaic Acquisition; Devices: Z, Y, X, Automatic Save (AS): ON; Laser power: 100%; Sheet width: 100%; Sheet NA: 0.109 (thickness 7 μm); Zoom: 0.8×; Light sheet: Right laser; Liquid: DBE; Dynamic Horizontal Focus (DHF): ON (16 steps); Start position: 0.000 μm, End position: −5500 μm, Range: −5500 μm, Stepsize: 5.000 μm, # Images: 1101; Exposure time: ∼200 ms. Click ‘Autosave settings’ and rename to the appropriate sample ID. Take the background image and click ‘Use background’. Start the measurement.

Using the autofluorescence channel (488 laser), specific imaging landmarks for Dorsal and Ventral imaging quadrants are used to align the brains for consistent imaging across individuals. Autofluorescence signal intensity should be approximately equal in all dimensions of the brain (Figure 2). For the Dorsal quadrant, align the midline of the brain 300 pixels away from the left imaging border. Find the Z-plane immediately before the optic tectum appears and then position the sample so the posterior end of the forebrain aligns with the bottom of the imaging border. Ensure there is no tissue missing throughout the whole Z-stack (Figures 2A–2C-F). For ventral imaging, align the midline at 300 pixels away from the left imaging border. Find the Z-plane where the anterior commissure is completely visible, and position the brain so the ventral (top) side of the septal nuclei are positioned on the horizontal crosshair (Figures 2B–2G–J). Set the imaging parameters as outlined in Figure 3. Ensure the mosaic is set as described in Step 21. As the working distance of the light sheet objective is 6 mm, we imaged in 5 μm increments across a distance of 5.5 mm, resulting in 1101 Z-planes. Based on the size of the pigeon brain, our imaging strategy allows complete imaging of the right hemisphere, as well as a 1.5 mm medial section of the left hemisphere.

**Figure 4:**
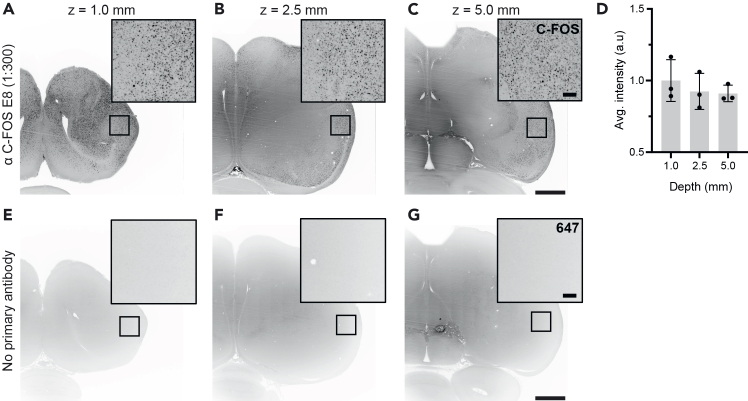
Light sheet detection of C-FOS positive cells Optical sections of a cleared pigeon brain stained for C-FOS at three different depths: (A) 1.0 mm, (B) 2.5 mm, (C) 5.0 mm. Insets in top right corner illustrate the equal intensity of C-FOS positive cells (black dots) observed after imaging. (D) Average intensity of detected cells (n=3 brains; 1 ROI per brain) after background removal. No C-FOS signal was observed in a negative control sample incubated with only the secondary antibody (Alexa-647, 1:300) at (E) 1.0 mm, (F) 2.5 mm and (G) 5.0 mm. Bars are plotted as a normalized average to the 1.0 mm intensity ± standard deviation. Scale bars: (C) 1.5 mm, inset: 200 μm. a.u: arbitrary units.

After staining, clearing, and imaging, C-FOS positive cells should be reliably and equally detected across the entire imaging depth (Figures 4A–4C). C-FOS signal intensity (n = 3 brains) was not significantly different between images taken 1 mm, 2.5 mm, or 5 mm deep into the tissue (one-way ANOVA; P=0.6196, F=0.5190; Figures 4D; Table S1). Importantly, no signal was observed throughout all three depths in a negative control sample that was incubated without primary antibody (Figures 4E–4G).

To analyze differential C-FOS levels in cleared brains after stimulation experiments, we recommend using the ClearMap analysis pipeline.^1^^,^^9^ Here, raw light sheet scans from all brains are first down-sampled and registered onto a reference template. C-FOS positive cells are then counted using a threshold-based spot detection algorithm, and an average heatmap of the C-FOS signal is created for each brain. Using these heatmaps, *t*-tests are performed on voxels throughout the entire brain. The output of this analysis is a three-dimensional p-value map that highlights regions with statistically significant differences in C-FOS levels between the control and treatment groups. As ClearMap was originally developed for the mouse brain, custom reference templates and atlases are needed for alternative model organisms. Our custom pigeon brain reference template is available at https://github.com/KeaysLab/ClearingProtocol. This reference template allows analyzing individual imaging tiles without the need for stitching. In the future, efforts should be directed to creating an annotated pigeon brain atlas compatible for ClearMap analyses.

## Limitations

This protocol is specifically designed for efficient clearing, immunolabeling of C-FOS, and light sheet imaging of the pigeon brain. For use in other avian species with differently sized brains, this procedure will need to be optimized. For example, clearing of an avian brain with a smaller volume will likely take less time to dehydrate, stain and clear. Additionally, a new imaging strategy and imaging landmarks will need to be determined reflecting neuroanatomical differences. The second notable limitation is that our protocol is optimized for the use of the C-FOS E-8 antibody. This marker has been successfully used in pigeons^1^ and chicken^16^^,^^17^ to detect C-FOS changes after sensory stimulation, and the target sequence is 100% conserved when comparing the human antigen and the pigeon C-FOS homologue (GenPept: XP_064919973.1). If working with another antibody^18^^,^^19^^,^^20^^,^^21^^,^^22^^,^^23^ or avian species, staining conditions will have to be adapted, and the specificity of the staining should be determined using positive control experiments. Thirdly, this protocol requires significant time investment. To cut down on the total protocol time, users could test alternative methanol/PBS dilution steps (e.g., 15–20% instead of 10%), optimize antibody incubation times (e.g., if superficial markers are employed), or use automated solution exchangers to increase the number of rinses that can be performed over 24 h.

## Troubleshooting

### Problem 1

Contamination in tissue (related to Steps 8 – 14).

As brain tissue is processed at 37°C for prolonged periods, conditions can promote the growth of microbials such as fungi or bacteria. Cloudy buffer solutions or fibril mold growths on or within the tissue are indicators of this problem. Contaminated brain tissue appears darker, with reduced antibody labeling in affected regions.

### Potential solution

Remove contaminated tissue from experimental analysis to avoid compromising the results. To minimize contamination risk, ensure solutions that are incubated at 37°C for over 1 day are filtered with a 0.2 μm filter and add 0.02% sodium azide. Only use sterile-filtered serum. Additionally, thoroughly clean the incubator with a strong disinfectant before each incubation. Ensure individual glass vials (for antibody incubations) are sealed tightly with parafilm.

### Problem 2

Bursting of brain tissue during dehydration or rehydration (related to Steps 6, 8, 15).

Rapid dehydration or rehydration of brain tissue can lead to rupture damage (particularly at the posterior end of the forebrain). Small lesions after rehydration can indicate this problem.

### Potential solution

Ensure dehydration and rehydration steps are completed with methanol diluted in 1× PBS, not H_2_0. Perform steps slowly (∼12 h each) and in small increments (10%). Be gentle when changing solutions to avoid mechanical damage. Tissue that was damaged during sample preparation is more likely to show distortion or bursts during dehydration.

### Problem 3

Primary or secondary antibody does not penetrate efficiently (related to Steps 11, 13).

Indicators of this problem are a complete lack of signal within the tissue, or non-uniform staining across the tissue.

### Potential solution

If a bright ring of staining is seen on the edge of the tissue and the signal decreases with tissue depth, decrease the primary antibody concentration. This could be a result of excess secondary binding to its primary target at the surface of the tissue which prevents further penetration. If little or no signal is seen, binding of the primary antibody needs to be improved. Make sure that the dura mater is fully removed during tissue dissection. Increase the primary antibody concentration, incubation time, and potentially permeabilization.

### Problem 4

Tissue browning and insufficient clearing (related to Step 16).

Brains may look brown, cloudy and/or not sufficiently transparent after the final DBE incubation.

### Potential solution

While some color change is expected during this procedure, excess browning is a sign of oxidation. To prevent this, use high quality organic solvents (as listed in the key resources table) and ensure tubes are fully filled during washes to prevent oxygenation (especially during DCM steps). DCM is ideally used fresh. Make sure to perform the final clearing steps (16c–f) quickly to reduce air exposure. If brains are cloudy or opaque, transfer the brains to fresh DBE and leave for 3-4 more days. Ensure there are no traces of water when completing the final refractive index matching.

### Problem 5

Poor imaging quality (related to Steps 17 – 26).

Final light-sheet images appear unfocused or dim.

### Potential solution

Before imaging, align the lasers of the microscope according to the manufacturer’s instructions. Ensure the imaging parameters are set as described above, particularly that the dynamic horizontal focus is on during C-FOS signal acquisition. Check the microscope stage is working mechanically in all axes and pay attention to any grinding sounds when the stage is moved. This could indicate a stage problem. Make sure the sample holder is secured and mounted evenly. Change the DBE in the imaging chamber regularly and remove any noticeable particles that could compromise the beam path.

## Resource availability

### Lead contact

Further information and requests for resources and reagents should be directed to and will be fulfilled by the lead contact, David A. Keays (keays@biologie.uni-muenchen.de).

### Technical contact

Technical questions on executing this protocol should be directed to and will be answered by the technical contacts, Spencer D. Balay (balay@bio.lmu.de) and Gregory C. Nordmann (gregory.nordmann@bi.mpg.de).

### Materials availability

• This study did not generate new unique reagents. Commercially available reagents are listed in the key resources table.
• 3D printer files for the polyetheretherketone pigeon brain sample holder and dorsal/ventral insert molds have been uploaded to the Keays Lab GitHub repository (https://github.com/KeaysLab/ClearingProtocol).
• Image files for the custom pigeon brain reference template have been uploaded to the Keays Lab GitHub repository (https://github.com/KeaysLab/ClearingProtocol).
• The GitHub repository DOI is as follows: https://doi.org/10.5281/zenodo.18406963.

### Data and code availability

• Raw data and statistics for the signal intensity experiment (Figure 4) are provided as a supplementary file.
• This study did not generate code.

## Acknowledgments

We thank the core facilities of the IMP Vienna and LMU Munich, the Graphics Department of the IMP Vienna for illustrations, the workshop of the IMP Vienna for the sample holder and brain mold, and all members of the Keays lab for discussions and feedback. Funding was provided by the 10.13039/100010663European Research Council (D.A.K., 336725 and 819336) and NSERC (S.D.B., PGSD 557761).

## Author contributions

Conceptualization, S.D.B., G.C.N., E.P.M., L.L., S.N., and D.A.K.; methodology, S.D.B., G.C.N., E.P.M., L.L., and S.N.; investigation, S.D.B., G.C.N., and S.N.; visualization, S.D.B. and G.C.N.; funding acquisition, D.A.K.; supervision, D.A.K.; writing – original draft, S.D.B., G.C.N., and D.A.K.; writing – review and editing, all authors.

## Declaration of interests

The authors declare no competing interests.

## Supplemental information

Table S1. C-FOS intensity measurements and statistics, related to Figure 4 (A) **“**Figure 4D_stats” sheet provides the average C-FOS intensity values normalized to the 1 mm average value and the statistical details of a One-way ANOVA with Tukey’s multiple comparisons test and a Shapiro-Wilk normality test. (B) “Figure 4D_raw” sheet provides the raw intensity values from all C-FOS positive cells detected in each depth (1 mm, 2.5 mm and 5 mm) from each bird (n = 3).
